# Supplementary material for: COVID-19 ICU and mechanical ventilation patient characteristics and outcomes—A systematic review and meta-analysis
Source: PLoS One. 2021 Feb 11;16(2):e0246318. doi: 10.1371/journal.pone.0246318 (PMC7877631; doi:10.1371/journal.pone.0246318)
Supplement: S2 Table — (PDF) [file pone.0246318.s002.pdf]

S2 Table. Results of the individual components of the quality assessement of the included studies

ID	1st Criterion	2nd Criterion	3rd Criterion	4th Criterion	5th Criterion	6th Criterion	7th Criterion	8th Criterion	9th Criterion	10th Criterion	11th Criterion	12th Criterion	13th Criterion	14th Criterion	Overall assessment
**China**															
Bi/April	Yes	Yes	NA	Yes	No	Yes	NM	No	Yes	No	Yes	NA	Yes	No	Good
Cao/May	Yes	Yes	NA	Yes	No	Yes	Yes	No	Yes	No	Yes	NA	Yes	No	Good
Du/April	Yes	Yes	NA	No	No	Yes	Yes	No	Yes	No	Yes	NA	Yes	Yes	Good
Feng/April	Yes	Yes	NA	Yes	No	Yes	NM	No	Yes	No	Yes	NA	Yes	Yes	Good
Guan/February	Yes	Yes	NA	Yes	No	Yes	NM	No	Yes	No	Yes	NA	Yes	No	Good
Huang/January	Yes	Yes	NA	Yes	No	Yes	Yes	No	Yes	No	Yes	NA	Yes	No	Good
Lapidus/April	Yes	No	NA	No	No	Yes	Yes	No	No	No	Yes	NA	No	No	Fair
Lei/April	Yes	Yes	NA	No	No	Yes	Yes	No	Yes	No	Yes	NA	Yes	No	Fair
Wang/April	Yes	Yes	NA	No	No	Yes	Yes	No	Yes	No	Yes	NA	Yes	Yes	Good
Xu/March	Yes	Yes	NA	No	No	Yes	Yes	No	Yes	No	Yes	NA	Yes	Yes	Good
Yang/February	Yes	Yes	NA	Yes	No	Yes	Yes	No	Yes	No	Yes	NA	Yes	Yes	Good
Zheng/April	Yes	No	NA	Yes	No	Yes	Yes	No	No	No	Yes	NA	No	No	Fair
Zhou/March	Yes	Yes	NA	Yes	Yes	Yes	Yes	No	Yes	No	Yes	NA	Yes	Yes	Good
**USA**															
Arentz/March	Yes	No	NA	Yes	No	Yes	No	No	Yes	No	Yes	NA	No	No	Fair
Argenziano/April	Yes	No	NA	Yes	No	Yes	Yes	No	Yes	No	Yes	NA	Yes	No	Good
Auld/April	Yes	Yes	NA	Yes	No	Yes	No	No	Yes	No	Yes	NA	Yes	No	Fair
Bhatraju/March	Yes	Yes	NA	Yes	No	Yes	Yes	No	Yes	No	Yes	NA	No	No	Good
Goyal/May	Yes	Yes	NA	Yes	No	Yes	NM	No	Yes	No	Yes	NA	No	No	Fair
Myers/April	Yes	No	NA	Yes	No	Yes	Yes	No	Yes	No	Yes	NA	Yes	No	Good
Paranjpe/April	Yes	Yes	NA	No	No	Yes	Yes	No	Yes	No	Yes	NA	Yes	Yes	Good
Richardson/April	Yes	Yes	NA	No	No	Yes	Yes	No	Yes	No	Yes	NA	No	Yes	Good

Ziehr/April	Yes	Yes	NA	No	No	Yes	Yes	No	Yes	No	Yes	NA	Yes	Yes	Good
**UK**															
ICNARC/May	Yes	Yes	NA	No	No	Yes	Yes	No	Yes	No	Yes	NA	Yes	Yes	Good
Docherty/April	Yes	No	NA	No	No	Yes	Yes	No	Yes	No	Yes	NA	No	Yes	Fair
**Mexico**															
Acosta/May	Yes	Yes	NA	Yes	No	Yes	NM	No	Yes	No	Yes	NA	Yes	No	Good
**Italy**															
Grasselli/April	Yes	Yes	NA	No	No	Yes	Yes	No	Yes	No	Yes	NA	No	Yes	Good
**Spain**															
Borobia/May	Yes	Yes	NA	Yes	No	Yes	Yes	No	Yes	No	Yes	NA	No	Yes	Good
**France**															
Simonnet/April	Yes	Yes	NA	No	No	Yes	Yes	No	Yes	No	Yes	NA	Yes	Yes	Good

Abbreviations: NM = not mentioned; NA = not appliacable.
Individual criteria 1-14 definitions as detailed in the table below

Criterion number	Definition
1st	Was the research question or objective in this paper clearly stated?
2nd	Was the study population clearly specified and defined?
3rd	Was the participation rate of eligible persons at least 50%?
4th	Were all the subjects selected or recruited from the same or similar populations (including the same time period)? Were inclusion and exclusion criteria for being in the study prespecified and applied uniformly to all participants?
5th	Was a sample size justification, power description, or variance and effect estimates provided?
6th	For the analyses in this paper, were the exposure(s) of interest measured prior to the outcome(s)being measured?
7th	Was the timeframe sufficient so that one could reasonably expect to see an association between exposure and outcome if it existed?
8th	For exposures that can vary in amount or level, did the study examine different levels of the exposure as related to the outcome (e.g., categories of exposure, or exposure measured as continuous variable)?
9th	Were the exposure measures (independent variables) clearly defined, valid, reliable, and implemented consistently across all study participants?
10th	Was the exposure(s) assessed more than once over time?
11th	Were the outcome measures (dependent variables) clearly defined, valid, reliable, and implemented consistently across all study participants?
12th	Were the outcome assessors blinded to the exposure status of participants?
13th	Was loss to follow-up after baseline 20% or less?
14th	Were key potential confounding variables measured and adjusted statistically for their impact on the relationship between exposure(s) and outcome(s)?
