# Supplementary material for: COVID-19 ICU and mechanical ventilation patient characteristics and outcomes—A systematic review and meta-analysis
Source: PLoS One. 2021 Feb 11;16(2):e0246318. doi: 10.1371/journal.pone.0246318 (PMC7877631; doi:10.1371/journal.pone.0246318)
Supplement: S1 File — (PDF) [file pone.0246318.s003.pdf]

S1 File. Electronic search strategy

Library/Database: PubMed

Date of Search: May 1, 2020

Search String: ("COVID-19" OR "SARS-Cov-2") AND ("ICU" or "ventilator" OR "mechanical ventilation" OR "critical care" OR "intensive care" OR "hospitalized" OR "outcomes")

# Hits: 1503

Library/Database: Scopus

Date of Search: May 1, 2020

Search String: Article title, Abstract, Keywords ("COVID-19" OR "SARS-Cov-2") AND ("ICU" or "ventilator" OR "mechanical ventilation" OR "critical care" OR "intensive care" OR "hospitalized" OR "outcomes")

# Hits: 1937

Library/Database: Virtual Health Library (VHL)

Date of Search: May 1, 2020

Search String: Title, Abstract, Subject ("COVID-19" OR "SARS-Cov-2") AND ("ICU" or "ventilator" OR "mechanical ventilation" OR "critical care" OR "intensive care" OR "hospitalized" OR "outcomes")

# Hits: 1716

Library/Database: medRvix & bioRvix

Date of Search: May 1, 2020

Search String: ("COVID-19" OR "SARS-Cov-2") AND ("ICU" or "ventilator" OR "mechanical ventilation" OR "critical care" OR "intensive care" OR "hospitalized" OR "outcomes")

# Hits: 2690
