# Supplementary material for: COVID-19 ICU and mechanical ventilation patient characteristics and outcomes—A systematic review and meta-analysis
Source: PLoS One. 2021 Feb 11;16(2):e0246318. doi: 10.1371/journal.pone.0246318 (PMC7877631; doi:10.1371/journal.pone.0246318)
Supplement: S2 File — (PDF) [file pone.0246318.s004.pdf]

National Institute for Health Research

UNIVERSITY of York
Centre for Reviews and Dissemination

Systematic review

1. * Review title.

Give the title of the review in English

Clinical epidemiology and outcome of ICU-admitted patients with or without mechanical ventilation in COVID-19 patients - a rapid systematic review and meta-analysis

2. Original language title.

For reviews in languages other than English, give the title in the original language. This will be displayed with the English language title.

3. * Anticipated or actual start date.

Give the date the systematic review started or is expected to start.

15/04/2020

4. * Anticipated completion date.

Give the date by which the review is expected to be completed.

30/06/2020

5. * Stage of review at time of this submission.

Tick the boxes to show which review tasks have been started and which have been completed. Update this field each time any amendments are made to a published record.

Reviews that have started data extraction (at the time of initial submission) are not eligible for inclusion in PROSPERO. If there is later evidence that incorrect status and/or completion date has been supplied, the published PROSPERO record will be marked as retracted.

This field uses answers to initial screening questions. It cannot be edited until after registration.

The review has not yet started: No

Review stage	Started	Completed
Preliminary searches	Yes	Yes
Piloting of the study selection process	Yes	Yes
Formal screening of search results against eligibility criteria	Yes	Yes
Data extraction	Yes	Yes
Risk of bias (quality) assessment	Yes	Yes
Data analysis	Yes	Yes

Provide any other relevant information about the stage of the review here.

6. * Named contact.

The named contact is the guarantor for the accuracy of the information in the register record. This may be any member of the review team.

Raymond Chang

Email salutation (e.g. "Dr Smith" or "Joanne") for correspondence:

Dr Chang

7. * Named contact email.

Give the electronic email address of the named contact.

rchang@meridianmedical.org

8. Named contact address

Give the full institutional/organisational postal address for the named contact.

102 E30th Street, New York, NY 10016 USA

9. Named contact phone number.

Give the telephone number for the named contact, including international dialling code.

+12126831221

10. * Organisational affiliation of the review.

Full title of the organisational affiliations for this review and website address if available. This field may be completed as 'None' if the review is not affiliated to any organisation.

Institute of East West Medicine

Organisation web address:

11. * Review team members and their organisational affiliations.

Give the personal details and the organisational affiliations of each member of the review team. Affiliation refers to groups or organisations to which review team members belong. **NOTE: email and country now**

MUST be entered for each person, unless you are amending a published record.

Dr Raymond Chang. Institute of East West Medicine
Dr Wei-zen Sun. National Taiwan University Hospital
Dr Khaled Elhusseiny. Faculty of Medicine, Al-Azhar University of Cairo

12. * Funding sources/sponsors.

Details of the individuals, organizations, groups, companies or other legal entities who have funded or sponsored the review.

None

Grant number(s)

State the funder, grant or award number and the date of award

13. * Conflicts of interest.

List actual or perceived conflicts of interest (financial or academic).

None

14. Collaborators.

Give the name and affiliation of any individuals or organisations who are working on the review but who are not listed as review team members. **NOTE: email and country must be completed for each person, unless you are amending a published record.**

15. * Review question.

State the review question(s) clearly and precisely. It may be appropriate to break very broad questions down into a series of related more specific questions. Questions may be framed or refined using PI(E)COS or similar where relevant.

What is the proportion and clinical characteristics and co-morbidities of ICU-admitted COVID-19 patients? What is the outcome of ICU-admitted COVID-19 patients? What is the length of stay of ICU-admitted COVID-19? What is the duration and outcome of mechanical ventilation in COVID-19 patients? What are possible determinants of outcome in ICU-admitted COVID-19 patients?

16. * Searches.

State the sources that will be searched (e.g. Medline). Give the search dates, and any restrictions (e.g. language or publication date). Do NOT enter the full search strategy (it may be provided as a link or attachment below.)

Pertinent studies will be identified independently by two researchers via a search of electronic databases MEDLINE, Scopus, EMBASE and the Cochrane Library, while preprints will be searched via the major preprint servers including bioRvix, medRvix, Preprints.org and Authorea, and additional official reports by government [e.g. Morbidity and Mortality Weekly Reports (MMWR) from the US CDC] and institutional agencies [e.g. Intensive Care National Audit and Research Center (ICNARC) of UK] and other relevant registries will be also be searched and included if they meet the inclusion criteria. Furthermore, relevant systematic reviews, or meta-analyses and bibliographic references from identified studies will be manually screened to identify additional studies. Study investigators or corresponding authors will be contacted to

clarify missing or unpublished data. The searches will be conducted without restrictions with date of search ranging from December 1st 2019 through May 1st 2020.

Search strategy combines relevant medical subject headings (MeSH) and keywords: ("COVID-19" OR "SARS-CoV-2" OR "SARS-CoV" OR "coronavirus") AND ("ventilator" OR "mechanical ventilation" OR "ventilation" OR "ventilated" OR "respiratory" OR "ICU" OR "critical care" OR "hospitalized" OR "outcomes".

Two researchers will independently conducted searches and will enlist an independent third reviewer if consensus cannot be reached as to inclusion or exclusion.

17. URL to search strategy.

Upload a file with your search strategy, or an example of a search strategy for a specific database, (including the keywords) in pdf or word format. In doing so you are consenting to the file being made publicly accessible. Or provide a URL or link to the strategy. Do NOT provide links to your search **results**.

Alternatively, upload your search strategy to CRD in pdf format. Please note that by doing so you are consenting to the file being made publicly accessible.

Do not make this file publicly available until the review is complete

18. * Condition or domain being studied.

Give a short description of the disease, condition or healthcare domain being studied in your systematic review.

Coronavirus disease 2019 (COVID-19) patients requiring hospitalization and subsequent mechanically ventilation due to respiratory failure.

19. * Participants/population.

Specify the participants or populations being studied in the review. The preferred format includes details of both inclusion and exclusion criteria.

Hospitalized patients with COVID-19 requiring mechanical ventilation.

20. * Intervention(s), exposure(s).

Give full and clear descriptions or definitions of the interventions or the exposures to be reviewed. The preferred format includes details of both inclusion and exclusion criteria.

Mechanical ventilation regardless of ventilator mode or setting.

21. * Comparator(s)/control.

Where relevant, give details of the alternatives against which the intervention/exposure will be compared (e.g. another intervention or a non-exposed control group). The preferred format includes details of both inclusion and exclusion criteria.

Hospitalized patients who are not mechanically ventilated.

22. * Types of study to be included.

Give details of the study designs (e.g. RCT) that are eligible for inclusion in the review. The preferred format includes both inclusion and exclusion criteria. If there are no restrictions on the types of study, this should be stated.

Official and national patient registry reports (e.g. the US CDC's MMWR reports or UK's Intensive Care

National Audit and Research Center), observational case series, prospective or retrospective cohort studies, case-control studies, randomized trials with patient outcome data. Animal studies or case reports will be excluded as well as studies "in progress" that are without outcomes data.

23. Context.

Give summary details of the setting or other relevant characteristics, which help define the inclusion or exclusion criteria.

Given that the COVID-19 pandemic lead to a surge of hospitalizations and ICU admissions world-wide and with a significant portion of patients requiring respirator support, it is imperative to further define the characteristics of COVID-19 patients requiring mechanical ventilation and the clinical efficacy of mechanical ventilation so as to further inform clinicians and policy makers as to its optimal deployment and to optimize the allocation of ventilator resources in this pandemic.

24. * Main outcome(s).

Give the pre-specified main (most important) outcomes of the review, including details of how the outcome is defined and measured and when these measurement are made, if these are part of the review inclusion criteria.

Duration (days) on mechanical ventilation, ICU and mechanical ventilation mortality rate.

* Measures of effect

Please specify the effect measure(s) for you main outcome(s) e.g. relative risks, odds ratios, risk difference, and/or 'number needed to treat.

Relative risks, odds ratios, risk difference

25. * Additional outcome(s).

List the pre-specified additional outcomes of the review, with a similar level of detail to that required for main outcomes. Where there are no additional outcomes please state ‘None’ or ‘Not applicable’ as appropriate to the review

Co-morbidities

* Measures of effect

Please specify the effect measure(s) for you additional outcome(s) e.g. relative risks, odds ratios, risk difference, and/or 'number needed to treat.

Relative risks, odds ratios, risk difference

26. * Data extraction (selection and coding).

Describe how studies will be selected for inclusion. State what data will be extracted or obtained. State how this will be done and recorded.

Data extraction will be conducted independently by two researchers using a standardized extraction form. Disagreements will be resolved through discussion or referral to an independent third reviewer. Initial criteria for inclusion/exclusion of studies will be based on PICOTS (i/ populations, ii/ interventions, iii/ comparators, iv/ outcomes, v/ timings and vi/ setting) elements specific to our study, namely i/ hospitalized patients irrespective of age diagnosed with COVID-19 who are placed on ii/ mechanical ventilation compared to those

who are iii/ not on mechanical ventilation, assessed for iv/ duration, complications of mechanical ventilation, ICU and hospital discharge, and mortality over v) any period of time. Data will only be considered for inclusion for patients with outcomes.

27. * Risk of bias (quality) assessment.

State which characteristics of the studies will be assessed and/or any formal risk of bias/quality assessment tools that will be used.

We will evaluate the risk of bias of each included study using predefined criteria. We will utilize the Newcastle-Ottawa scale or ROBINS-I for non-randomized studies dependent on number of studies identified, and the Cochrane Collaboration’s Risk of Bias tool for randomized controlled trials. If there are at least ten relevant studies with robust designs, a visual asymmetry assessment using funnel plot will be generated to detect publication bias. Additional criteria will be adopted from other quality appraisal tools if deemed appropriate.

28. * Strategy for data synthesis.

Describe the methods you plan to use to synthesise data. This **must not be generic text** but should be **specific to your review** and describe how the proposed approach will be applied to your data. If meta-analysis is planned, describe the models to be used, methods to explore statistical heterogeneity, and software package to be used.

We will narratively synthesize study and patient clinical parameters and outcomes reported by included studies in tabular form. All patient clinical information, including outcomes, will be pooled using frequencies, percentages or proportions, and means (± SDs). The heterogeneity between included studies will be assessed using both the $?^2$ test for homogeneity and I^2 statistic where appropriate. RevMan 5.3 will be used for data analysis. Outcomes will be assessed using Risk Ratios, with 95% confidence intervals (CI's). We will determine whether meta-analysis is appropriate to quantitatively summarize study findings based on heterogeneity of the studies. Given the likelihood that most studies identified would be case series or retrospective cohorts with sizeable variations amongst them (different country, settings and different populations, different healthcare systems with different routines of care, different populations), we will likely use a random effect model for meta-analysis to calculate pooled estimates with 95% CI for each outcome measure and Forest plots will be constructed to illustrate a summary of findings from individual prognostic studies.

29. * Analysis of subgroups or subsets.

State any planned investigation of ‘subgroups’. Be clear and specific about which type of study or participant will be included in each group or covariate investigated. State the planned analytic approach.

If adequate data pertaining to patient age, sex, co-morbidities, ventilator modes and settings, complications and other relevant outcomes are available, the review investigators plan to carry out a priori subgroup analyses between studies to explain any inconsistencies between important subgroups.

30. * Type and method of review.

Select the type of review, review method and health area from the lists below.

Type of review

Cost effectiveness
No

Diagnostic
No

Epidemiologic
No

Individual patient data (IPD) meta-analysis
No

Intervention
Yes

Meta-analysis
Yes

Methodology
No

Narrative synthesis
No

Network meta-analysis
No

Pre-clinical
No

Prevention
No

Prognostic
No

Prospective meta-analysis (PMA)
No

Review of reviews
No

Service delivery
No

Synthesis of qualitative studies
No

Systematic review
Yes

Other
No

Health area of the review

Alcohol/substance misuse/abuse

No

Blood and immune system
No

Cancer
No

Cardiovascular
No

Care of the elderly
No

Child health
No

Complementary therapies
No

COVID-19
Yes

For COVID-19 registrations please tick all categories that apply. Doing so will enable your record to appear in area-specific searches

Chinese medicine
Diagnosis
Epidemiological
Genetics
Health impacts
Immunity
Long COVID
Mental health
PPE
Prognosis
Public health intervention
Rehabilitation
Service delivery
Transmission
Treatments
Vaccines
Other

Crime and justice
No

Dental
No

Digestive system
No

Ear, nose and throat
No

Education
No

Endocrine and metabolic disorders
No

Eye disorders

No

General interest
No

Genetics
No

Health inequalities/health equity
No

Infections and infestations
Yes

International development
No

Mental health and behavioural conditions
No

Musculoskeletal
No

Neurological
No

Nursing
No

Obstetrics and gynaecology
No

Oral health
No

Palliative care
No

Perioperative care
No

Physiotherapy
No

Pregnancy and childbirth
No

Public health (including social determinants of health)
No

Rehabilitation
No

Respiratory disorders
Yes

Service delivery
No

Skin disorders
No

Social care
No

Surgery
No

Tropical Medicine
No

Urological
No

Wounds, injuries and accidents
No

Violence and abuse
No

31. Language.

Select each language individually to add it to the list below, use the bin icon to remove any added in error.
English

There is not an English language summary

32. * Country.

Select the country in which the review is being carried out. For multi-national collaborations select all the countries involved.

United States of America

33. Other registration details.

Name any other organisation where the systematic review title or protocol is registered (e.g. Campbell, or The Joanna Briggs Institute) together with any unique identification number assigned by them. If extracted data will be stored and made available through a repository such as the Systematic Review Data Repository (SRDR), details and a link should be included here. If none, leave blank.

34. Reference and/or URL for published protocol.

If the protocol for this review is published provide details (authors, title and journal details, preferably in Vancouver format)

Add web link to the published protocol.

Or, upload your published protocol here in pdf format. Note that the upload will be publicly accessible.

Yes I give permission for this file to be made publicly available

Please note that the information required in the PROSPERO registration form must be completed in full even if access to a protocol is given.

35. Dissemination plans.

Do you intend to publish the review on completion?

Yes

Give brief details of plans for communicating review findings.?

We intend to communicate the progress and results of the review via initial publication of a manuscript on a preprint server (e.g. medRxiv) and subsequently submit the manuscript for peer-reviewed scientific journal

publication.

36. Keywords.

Give words or phrases that best describe the review. Separate keywords with a semicolon or new line. Keywords help PROSPERO users find your review (keywords do not appear in the public record but are included in searches). Be as specific and precise as possible. Avoid acronyms and abbreviations unless these are in wide use.

COVID-19, Sars-CoV-2, ventilator, mechanical ventilation, respiratory failure, ICU, pneumonia

37. Details of any existing review of the same topic by the same authors.

If you are registering an update of an existing review give details of the earlier versions and include a full bibliographic reference, if available.

38. * Current review status.

Update review status when the review is completed and when it is published.New registrations must be ongoing so this field is not editable for initial submission.
Please provide anticipated publication date

Review_Completed_not_published

39. Any additional information.

Provide any other information relevant to the registration of this review.

* We inititally enter a project formal start date of May 1st, but since we actually started scouting the literature with some preliminary searches earlier on Apr 15th to form a feel of the scope of the project, we now changed to start date to Apr 15th.

* Upon a search for related or similar systematic reviews or meta-analyses already registered, we identifed 2 protocols addressing the similar field: one protocol (CRD42020178187) addresses non-invasive ventilation used in respiratory support of COVID-19 patients, which would be a different intervention from the invasive mechanical ventilation we intend to examine. The other related protocol (CRD42020181267) is quite different in scope from ours. It is a systematic review which addresses a single focused question "What is the chance of survival after being intubated" in COVID-19 patients. The target population overlaps with our protocol but it is only concerned with "mortality" as the only measured outcome whereas our protocol is also intent to examine the clinical epidemiology of mechanically ventilated patients with COVID-19. That protocol also employs a much narrower search field (research papers of observational studies only) which would not include a large number of patients not reported in research papers per se but only as raw data in clinical registries (e.g. UK's "Intensive Care National Audit and Research Center" or ICNARC), and we thus believe that our proposed review is distinct enough in scope and focus to be considered and registered separately and independently.

40. Details of final report/publication(s) or preprints if available.

Leave empty until publication details are available OR you have a link to a preprint (NOTE: this field is not editable for initial submission). List authors, title and journal details preferably in Vancouver format.

Give the link to the published review or preprint.
